# Ammonia volatization from conventional and stabilized fertilizers, agronomic aspects and microbiological attributes in a Brazilian coffee crop system

**DOI:** 10.3389/fpls.2023.1291662

**Published:** 2023-12-08

**Authors:** Leonardo Fernandes Sarkis, Mateus Portes Dutra, Damiany Pádua Oliveira, Tales Jesus Fernandes, Thaís Regina de Souza, Victor Ramirez Builes, Douglas Guelfi

**Affiliations:** ^1^ Department of Soil Science, Federal University of Lavras, Lavras, MG, Brazil; ^2^ Department of Statistics, Federal University of Lavras, Lavras, MG, Brazil; ^3^ Yara International, Berlin, Germany

**Keywords:** nitrogen fertilizers, urease inhibitor, coffea arabica, basal respiration, enzymatic activity, soil health

## Abstract

We aimed to quantify the N losses through volatilization of the main conventional and stabilized N fertilizers applied in coffee plantations. Additionally, we also assessed microbiological attributes of the soil (microbial biomass carbon (MBC); microbial biomass nitrogen (MBN); microbial basal respiration (MBR); metabolic quotient (qCO_2_); urease, β-glucosidase, acid phosphatase, and arylsulfatase activities) and agronomic aspects of the crop (N content in the leaves and beans, yield, and N exportation by the beans). Treatments consisted of the combination of three fertilizers (ammonium nitrate - AN, conventional urea - U, and urea with N- (n-butyl) thiophosphoric triamide (NBPT) - U_NBPT_, and five doses of N (0, 150, 275, 400, and 525 kg ha^-1^ year^-1^ of N), with four replicates, totalling 60 experimental plots. In the two crop seasons evaluated, daily and cumulative losses of N-NH_3_ from the split fertilizer applications were influenced by the N fertilizer technologies. The application of U resulted in losses of 22.0% and 22.8% for the doses of 150 and 400 kg ha^-1^ year^-1^ of N. This means that 66 and 182 kg ha^-1^ of N-NH_3_ were lost, respectively, at the end of six fertilizations with U. U_NBPT_ reduced urease activity and N-NH_3_ losses compared to conventional urea, avoiding the volatilization of 15.9 and 24.3 kg ha^-1^ of N. As for AN, N-NH_3_ losses did not exceed 1% of the applied dose, regardless of the weather conditions during the fertilization. Urease activity was higher on days of maximum NH_3_ volatilization. There was an effect of the N sources (NS), soil sampling time (ST), and their interaction (NS × ST) on the MBN and arylsulfatase activity. The N sources also influenced the MBC and the qCO_2_. A substantial amount of N was removed from the system by the beans and husks of the harvested fruits. Our study showed that N fertilizer technologies are interesting options to reduce N-NH_3_ losses by volatilization, increase N retention in the soil, and improve microbiological attributes and the sustainability of coffee production systems.

## Graphical Abstract

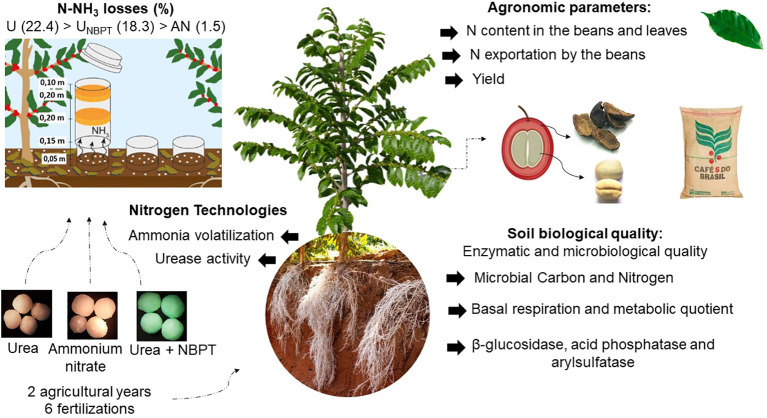

## Highlights

- Microbiological indicators reflect long-term soil coffee management.
- N-fertilization increases the carbon input and the microbiological activity of the system.
- U_NBPT_ reduces urease activity and N-NH_3_ losses.
- N-NH_3_ losses by ammonium nitrate do not exceed 1%, regardless of weather conditions.
- The high soil biological quality depends on the correct management of fertilization.

## 1 Introduction

Brazil is the world’s largest producer and exporter of coffee (*Coffea arabica* L.). Increasing fertilizer use efficiency through innovative technologies reduces costs of production, improves the quality of the beverage, and makes farming activity more sustainable and competitive, especially in times of high input prices. The application of nitrogen (N) fertilizers is essential for the productivity of this high-value crop. Brazilian agriculture consumed 9.2 million tonnes of N fertilizers in 2020 (FAOSTAT, 2021), in which coffee production alone was estimated do consume 10-15% of the country’s N (1.4 million tonnes per year).

The intense dynamics of the N transformations in the soil and the diverse pathways it can be lost in coffee production systems result in low N fertilizer use efficiency (NUE) (Salamanca-Jimenez et al., 2017). Studies using ^15^N demonstrated that coffee plants recover less than 25% of the N fertilizer when conventional urea is used (Cannavo et al., 2013; Salamanca-Jimenez et al., 2017), a complex scenario considering that approximately 50% of the global N fertilizer production is represented by urea (Heffer and Prud’homme, 2016; FAOSTAT, 2021).

Ammonia (NH_3_) volatilization is the main process of N loss in coffee cultivation areas in Brazil, especially when conventional urea is applied without the incorporation and in the presence of plant residues (Martins et al., 2021; Freitas et al., 2022; de Souza et al., 2023), common practices in perennial crops such as coffee. Generally, for every three nitrogen applications with conventional urea in coffee systems, one is lost due to volatilization (Chagas et al., 2016; Dominghetti et al., 2016; Freitas et al., 2022; de Souza et al., 2023), configuring the most important agronomic loss.

The enhanced-efficiency fertilizers (EEFs) encompass many technologies for fertilizers and its one of the most studied strategies to increase NUE (Chien et al., 2009; Azeem et al., 2014; Timilsena et al., 2015) and mitigate greenhouse gas emissions in agriculture. For N fertilizers, these technologies are divided in the following main categories: conventional fertilizers, stabilized fertilizers, slow-release fertilizers, controlled-release fertilizers, and their blends (Guelfi, 2017). The proper use of EEFs is an interesting option to reduce N losses, carbon footprint, and climate change, in addition to increasing N retention in the soil (Snyder, 2017; Zhang et al., 2021; Freitas et al., 2022).

Stabilized fertilizers have additives, such as urease inhibitors (NBPT, NPPT, 2-NPT, and Duromide) and nitrification inhibitors (DCD, DMPP, and DMPSA), that inhibit or delay a determined stage of the N transformation process in the soil (Guelfi, 2017; Byrne et al., 2020). In Brazilian coffee cultivation, urease inhibitors are already widely used, while nitrification inhibitors or the combination of these two subgroups are still not significant in the national market. Currently, some authors have highlighted the need to evaluate the possible negative impacts of some additives on human health in future studies (Cai and Akiyama, 2017), which may be related to the persistence of some molecules in the environment.

The N-(n-butyl)thiophosphoric triamide (NBPT) is currently the most widely used molecule as a urease inhibitor (Sanz-Cobena et al., 2011). NBPT is reported to effectively reduce N-NH_3_ losses through volatilization when added to urea (Adotey et al., 2017; Santos et al., 2020). The reduction of NH_3_ losses is related to the maintenance of the N in the form of amide through a temporary inhibition of the urease activity, the enzyme which catalyzes the hydrolysis of urea (Santos et al., 2021; Freitas et al., 2022). This is due to the ability of the NBPT to oxidize into its analog, NBPTO, which forms stable complexes with the enzyme, causing its inactivation (Sanz-Cobena et al., 2011). The potential inhibition of the additive depends on the concentration and stability of the inhibitor in the fertilizer, which can vary depending on its compatibility with physical mixtures, time, and storage temperature.

The use of stabilized fertilizers may not be the most suitable technology among the strategies to reduce N-NH_3_ losses in a brazilian coffee crop system, as they depend on the occurrence of rain to incorporate the N fertilizer. The highest N losses through volatilization for conventional urea occur in the first 7 days after fertilization (Costa et al., 2003; Afshar et al., 2018), which is a critical period of losses (CPL). NBPT typically delays the CPL to increase the chance of incorporation of the N fertilizer into the soil by the precipitation. However, relying solely on weather conditions represents a high risk of inefficiency in N fertilizer incorporation, as it also depends on both the volume and intensity of rainfall (Dawar et al., 2011). In addition, the architecture of the coffee plants creates a barrier that reduces the rainfall under the canopy and the direct incidence on the N fertilizer applied in that location.

Currently, microbiological attributes have been used and disseminated as a tool to ensure ecosystem sustainability and monitoring the health of soil studies. The attributes most used as “microbiological indicators of soil quality” are microbial biomass carbon, microbial biomass nitrogen, microbial basal respiration, and enzymatic activities (Doran and Parkin 1994; Dick et al., 1996). The enzymes most used for this purpose are related to the C, N, P, and S cycles (Gil-Sotres et al., 2005; Moreira and Siqueira, 2006; Bastida et al., 2008).

Our hypothesis was that the technologies for nitrogen fertilizers can help mitigate N losses and improve agronomic efficiency and microbiological attributes in coffee fields. Therefore, the aims of this study were: i) quantify N-NH_3_ losses from two conventional sources of N and from urea stabilized with NBPT at two doses of N (150 and 400 kg ha^-1^); ii) determine the effect of nitrogen fertilizer technologies and doses of N (0, 150, 275, 400, and 525 kg ha^-1^ crop season^-1^, divided into three applications) for improvement in the nutrition and yield of coffee plants; iii) update the numbers of N exportation by the beans of recent coffee cultivars and understand the real N demand to avoid nitrate losses by leaching; iv) evaluate the effect of nitrogen fertilization on soil microbiological attributes (microbial biomass carbon, microbial biomass nitrogen, microbial basal respiration, metabolic quotient, urease, β-glucosidase, acid phosphatase and arylsulfatase enzyme activities; and, finally, v) support researchers, commercial farmers and fertilizer industries with a new insight and inedited results of N fertilizer technologies as interesting options for reducing N-NH_3_ losses by volatilization and increasing the sustainability of coffee production systems.

## 2 Materials and methods

### 2.1 Characterization of the experimental area

**Figure 1:**
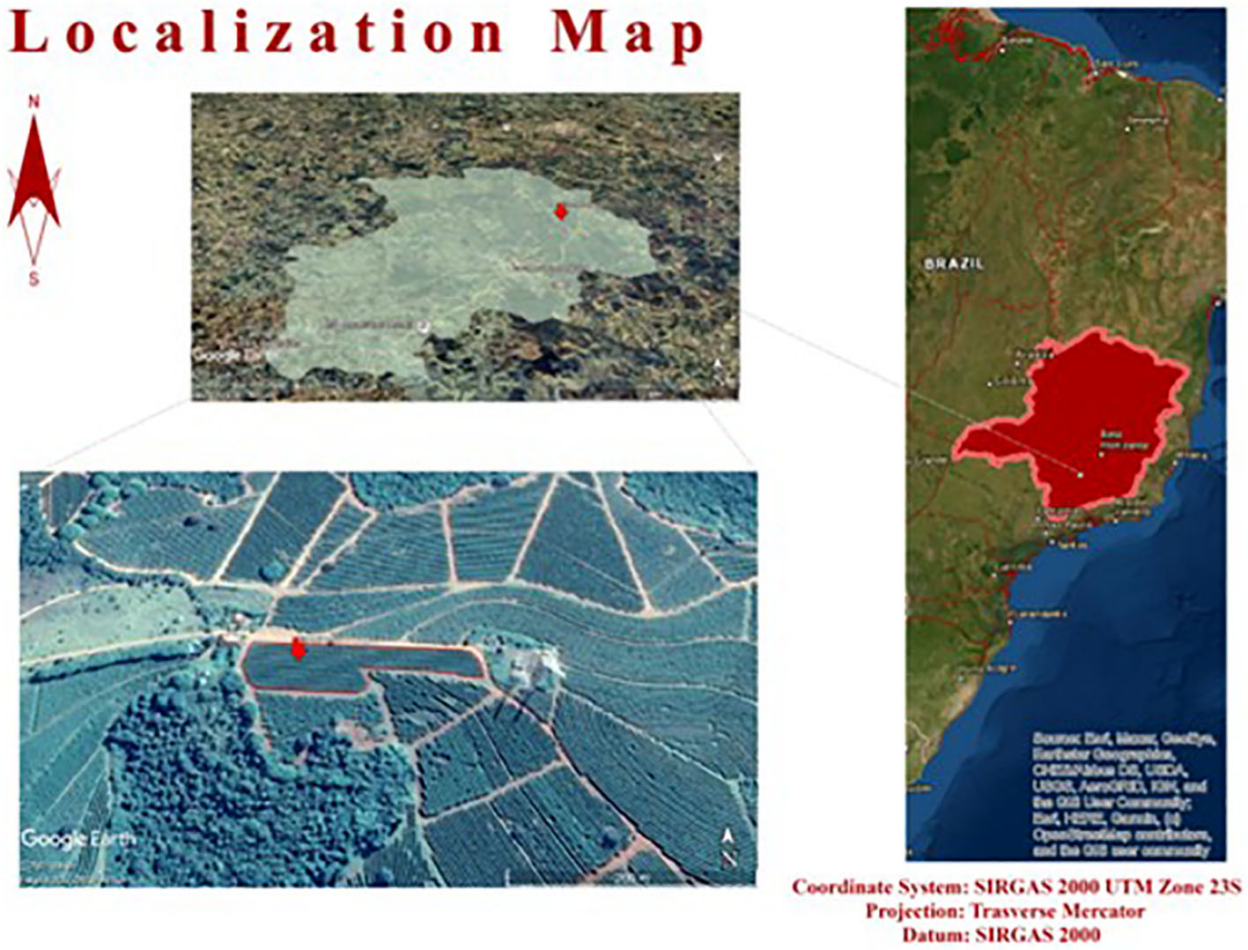
Location of the experimental areas in the municipality of Santo Antônio do Amparo, Minas Gerais state, Brazil.

The experiment was conducted during the 2019/2020 and 2020/2021 crop seasons with coffee plants under field conditions. The commercial plantation belongs to the Lagoa Coffee Plantation, owned by the NKG/Fazendas Brasileiras group, located in the municipality of Santo Antônio do Amparo, in the state of Minas Gerais, Brazil (**Figure 1**).

This municipality is located in a traditional coffee-producing region in Brazil within the Campos das Vertentes geographical indication, at 1,100 meters of altitude, latitude 20°53’26.04” S, and longitude 44°52’04.14” W. According to the Köppen classification, the climate is Cwa, with a humid tropical climate with a dry winter and a temperate summer. The mean annual precipitation is approximately 1,493 mm and the mean annual temperature is 19.6°C.

The soil was classified as an Oxisol according to United States Department of Agriculture (USDA) Soil Taxonomy (Soil Survey Staff, 2018). Before the experiment, soil samples were collected at 0 to 20 cm depth to identify the texture as 32%, 8%, and 60% of sand, silt, and clay, respectively, using the Bouyoucos method (Bouyoucos, 1951).

The five years old plantation in the production phase was planted with *Coffea arabica* L., Catuaí Vermelho – IAC 99 cultivar. Plants were spaced by 3.40 m between rows and 0.6 m between plants, resulting in 4,902 plants ha^-1^.

### 2.2 Fertilizer characterization

The fertilizers used in this study were chosen from the main technologies currently used in Brazilian coffee production. These fertilizers belong to two major technology groups: conventional and stabilized. All fertilizers were photographed using an Olympus microscope, SZ60 Japan model (S.1). For the conventional group, U (46% N) (S.1a) and AN (33% N) (S.1b) were used. For the stabilized group, U_NBPT_ (46% N) (S.1c) was used.

The concentration of NBPT in the fertilizers was analyzed by liquid chromatography (HP1100 Agilent model) with a diode array detector, according to the method described by the European Committee for Standardization (2008). This procedure was done to verify if the concentration of the additive in U_NBPT_ was the same as the manufacturer’s specification (530 mg kg^-1^ of NBPT). However, at the time of application, the NBPT concentration was 110 mg kg^-1^, indicating that there was degradation of the molecule between the manufacturing and commercialization. Some conditions such as storage time and temperature (Cantarella et al., 2018) and contact with phosphate fertilizers and ammonium sulfate that contain free acidity (Sha et al., 2020) accelerate the degradation of this additive and, consequently, reduce the potential for enzyme inhibition.

The treatment of urea with this additive consists of spraying 2.5 to 3 L of a 20% NBPT solution per t^-1^ of fertilizer. This solution is composed of the NBPT molecule, organic solvent, and dye used to distinguish the stabilized from the conventional fertilizers.

### 2.3 Experimental design

**Figure 2:**
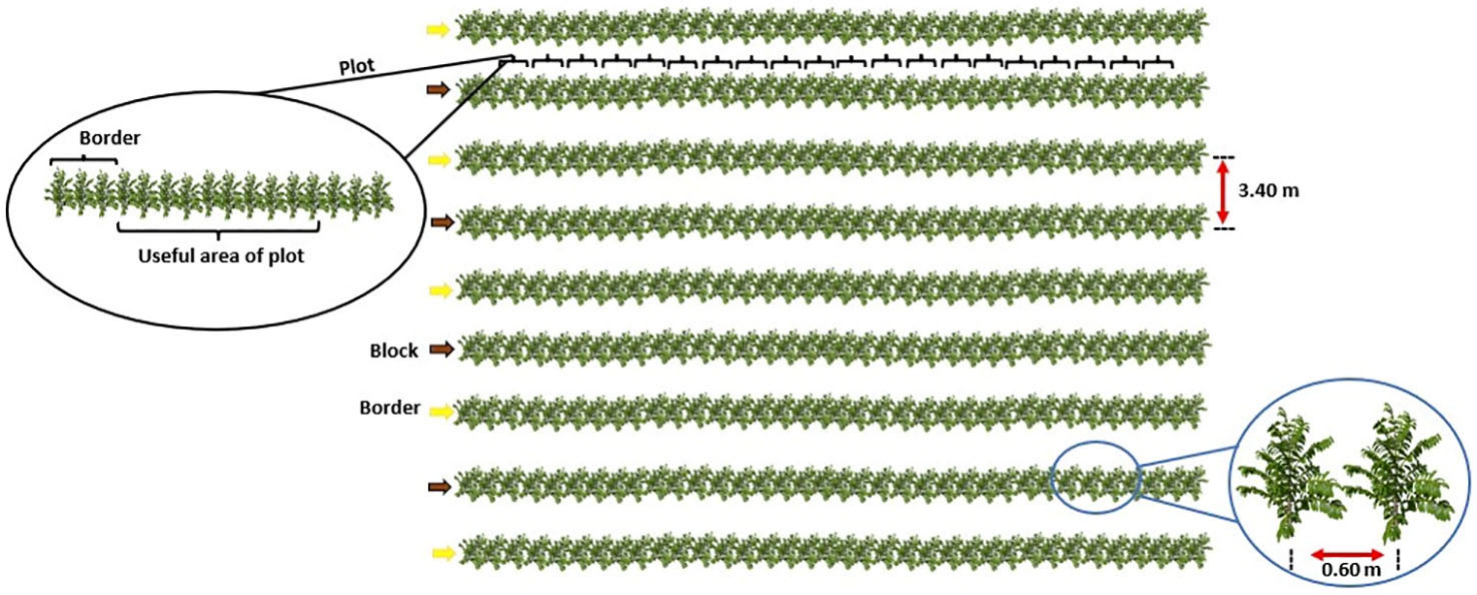
Illustration of the experimental design.

A randomized block design was used in a 3 × 5 factorial scheme, composed of three fertilizers and five doses with four replications, totaling 60 experimental units. Each experimental unit was composed of 16 coffee plants. The area comprising the central 10 plants was used as the useful area for data collection. An entire row on each site was left as border between blocks (**Figure 2**).

The treatments were composed of U, AN, and U_NBPT_, applied at doses of 0, 150, 275, 400, and 525 kg ha^-1^ year^-1^ of N. The fertilization was split into three equal parts applied 30 to 40 days after the previous fertilization, starting in November, at the beginning of the rainy season. All fertilizers were applied as topdressing and under the projection of the canopy of the coffee plants.

### 2.4 Complementary fertilization management

Liming was done 60 days before the application of the treatments to increase soil base saturation to 60%. Each treatment plot received 1.1 t ha^-1^ of limestone (90% ENP 16% MgO), 1.5 t ha^-1^ of gypsum, and 400 kg ha^-1^ of magnesium oxide (70% MgO). Additional fertilization was done with KCl (60% K_2_O) at 260 kg ha^-1^ year^-1^ of K_2_O divided into three applications and triple superphosphate (TSP – 46% P_2_O_5_) at 100 kg P_2_O_5_ ha^-1^ year^-1^ applied in a single application. Both KCl and TSP were applied in the projection of the coffee tree canopy.

Micronutrients were applied via foliar fertilization along with the plant disease control treatments during the formation and management of the plantation. A commercial product with the following composition was applied at 5 kg ha^-1^ in 300 L ha^-1^ of solution: 6.0% of zinc (ZnSO_4_), 3.0% of boron (H_3_BO_3_), 2.0% of manganese (MnSO_4_), 10% of copper (Cu(OH)_2_), 10% of sulfur (SO_4_), 1.0% of magnesium (MgSO_4_) and 10% of potassium (KCl). This fertilization was divided into three applications per year at intervals of 45 days between November and February. The soil was also fertilized with 3 kg ha^-1^ of B as H_3_BO_4_ and 5 kg ha^-1^ of Zn as ZnSO_4_ along with the first application of the maintenance fertilization.

### 2.5 Monitoring of climatic conditions

A climatological station set up near the experimental area was used for daily monitoring of rainfall, temperature (maximum and minimum), and relative humidity throughout the period of conducting the trials in the four agricultural crop seasons. Rain collectors were also installed beneath the coffee tree canopy to assess how much these plants reduce the direct incidence of rain where limestone and fertilizer are applied.

### 2.6 Quantification of the N-NH_3_ losses

**Figure 3:**
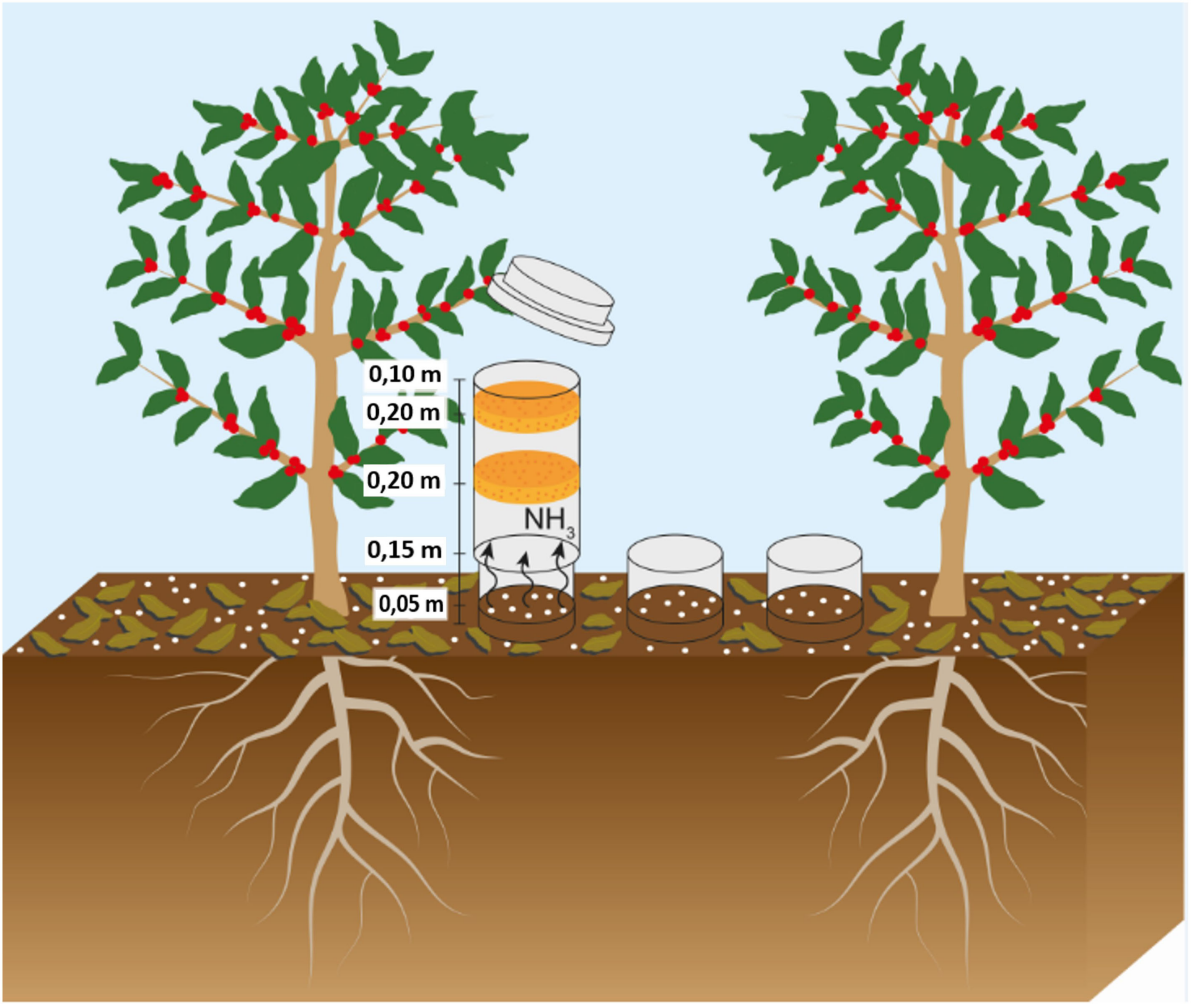
Illustration of the collectors used for the quantification of ammonia emissions.

A 3 × 2 factorial arrangement was adopted involving the same plots with the N sources of experimental design (described above) and two of those doses of N (150 and 400 kg N ha^-1^ crop season^-1^) were selected. The losses of N through NH_3_ volatilization resulting from soil N fertilization were quantified using the semi-open collector, adapted from Lara-Cabezas et al. (1999) method for capturing N-NH_3_. In each experimental plot that received 150 and 400 kg ha^-1^ of N, three 200 mm diameter bases made of PVC, with 20 cm height, were installed at 5 cm depth in the soil under the projection of the canopies (**Figure 3**).

PVC chambers similar to the bases but measuring 50 cm height were used to collect the emissions. Specifications are presented in **Figure 3**. Within each base (0.2 linear meters), the amount of fertilizer corresponding to the dose applied per hectare was added.

Immediately after fertilization, collection chambers were added over the bases in all plots. Within each chamber, two laminated foam disks with 0.02 g cm^-3^ density and 2 cm thickness, cut to the same diameter as the tube, were inserted. The disks were positioned 30 and 40 cm above the soil. The lower sponge was previously soaked with 80 ml of a solution composed of H_3_PO_4_ (60 ml L^-1^) and glycerin (50 ml L^-1^) to retain the volatilized ammonia. The upper sponge served to protect against possible environmental contamination.

The lower sponges were collected on the 1^st^, 2^nd^, 3^rd^, 4^th^, 5^th^, 6^th^, 8^th^, 10^th^, 13^th^, 16^th^, 20^th^, 24^th^, 28^th^, and 32^nd^ day after each fertilizer application. After replacing the sponges, the chamber was switched to the other base to reduce the spatial variability of the ammonia emissions. This rotation allowed a greater influence of climatic variations, such as temperature and precipitation.

The solution contained in the sponge was then extracted by filtration through a Buchner funnel and a vacuum pump, along with five consecutive washes with 80 mL of distilled water. From the extract, 20 mL were taken to determine the N content by distillation using the Kjeldahl (1883) method. The N content in the sample was calculated according to Equation 1:


NT=[(Va–Vb)×F×0.1×0.014×100]/P1

in which TN: Total nitrogen concentration in the sample (%); Va: Volume of hydrochloric acid solution used for titration of the sample (mL); Vb: Volume of hydrochloric acid solution used for titration of the blank (mL); F: Correction factor for 0.01 M hydrochloric acid; P1: mass of the sample (g).

The N concentration in the samples collected in the area of the chambers was extrapolated to the percentage of N-NH_3_ loss per hectare. To calculate the accumulated losses during the evaluation days, the losses from the 1^st^ and 2^nd^ day were added, then the sum of these was added to the 3rd day, and so on until the last day of sampling.

### 2.7 Microbiological analyses

#### 2.7.1 Soil sampling

To quantification of urease activity, soil samples were collected at 2 cm depth from the line of the N fertilizer application on the same dates that the sponges used to capture N-NH_3_ were collected. A split-plot design with repeated measures over time was adopted with four replications. The soil, with the same field moisture, was sieved through a 2 mm mesh to remove plant debris, and then stored at 4°C until analysis.

To evaluate the effects of the N sources on microbial C and N, basal respiration and metabolic quotient, β-glucosidase, acid phosphatase, and arylsulfatase, a split-plot design with repeated measures over time was also adopted with four replications. The design consisted of two sampling times in plots with no N (dose 0) and in plots with 400 kg ha^-1^ of N of AN, U, and U_NBPT_ sources to assess the impact of this dose, often used by farmers, on microbiological attributes in productive and high-investment coffee plantation. Soil samples from the 0-0.10 m layer were collected in the 2021/2022 crop season, both in the projection of the coffee tree canopy and in the fertilization line of each plot. The samples were collected two times: immediately before the start of the N fertilization and after 2/3 (267 kg N ha^-1^) of the N application to evaluate the influence of treatments that have been applied for consecutive years. The samples (2000 g each) were sieved through a 2 mm mesh and stored at 4°C for the following analyses. Other fertilization and management practices were kept constants for all plots, following the same detailed procedure as previously described.

#### 2.7.2 Microbial C and N

Microbial biomass carbon (MBC) and microbial biomass nitrogen (MBN) in the soil were determined in triplicate by the irradiation/extraction method (Islam and Weil, 1998) using 20 g of soil and 50 mL of potassium sulfate (0.5 mol L^-1^). The determination of MBC was carried out through oxidation with potassium dichromate (0.066 mol L^-1^) and subsequent addition of H_2_SO_4_ and H_3_PO_4_. The resulting mixture was heated on a hot plate until ebullition. After cooling, the solution was titrated with ammonium ferrous sulfate (0.033 mol L^-1^) and 1% diphenylamine as an indicator (Vance et al., 1987). MBN was determined by the Kjeldahl method.

#### 2.7.3 Basal respiration and metabolic quotient

The soil basal respiration (SBR) was quantified by incubating 20 g of soil in a hermetically closed container with NaOH solution (0.5 mol L^-1^) for three days at 28°C. The reaction was stopped by adding BaCl_2_.2H_2_O (0.5 mol L^-1^) and the solution was titrated with HCl (0.5 mol L^-1^) (Anderson and Domsch, 1993). The metabolic quotient (qCO_2_) was calculated by the division SBR/MBC (Anderson and Domsch, 1993).

#### 2.7.4 β-glucosidase, acid phosphatase, and arylsulfatase

Samples of 1 g of soil, containing 1 mL of substrates (ρ-nitrophenyl-β-D-glucoside for β-glucosidase, ρ-nitrophenyl-phosphate for acid phosphatase and ρ-nitrophenyl-sulfate for arylsulfatase) were incubated for one hour at 37°C in the presence of toluene and standard solution at specific pH for each activity (pH 6.0 for β-glucosidase, 6.5 for acid phosphatase and 5.8 for arylsulfatase). After the incubation period, the reaction was stopped with the addition of CaCl_2_ (0.5 mol L^−1^) and NaOH (0.5 mol L^−1^). The supernatant was filtered and readings were taken in a spectrophotometer at 410 nm by the colorimetric determination of the ρ-nitrophenol using 4-nitrophenyl β-D-glucopyranoside, potassium 4-nitrophenyl sulfate, and disodium 4-nitrophenyl orthophosphate as substrates, respectively (Dick et al., 1996; Aragão et al., 2020). The values of each activity were expressed in µg PNP g^-1^ soil h^-1^.

#### 2.7.5 Urease activity

The quantification of the urease activity was based on the determination of the N-NH_4_^+^ released after soil incubation with urea solution (Tabatabai and Bremner, 1972). For each treatment, a 5 g soil sample was mixed with 0.2 mL of toluene, 9.0 mL of buffer solution (pH 9.0), and 1.0 mL of a 0.2 mol L^-1^ urea solution, and then incubated for 2 hours at 25 °C in a temperature and humidity controlled chamber (model TE-371 TECNAL). After incubation, 35 mL of a 100 ppm solution composed of 2 mol L^-1^ KCl and 2 mol L^-1^ Ag_2_SO_4_ were added to stop the reaction. After shaking, the solution was left to rest for 5 minutes at room temperature, and the volume was then completed to 50 mL with KCl and Ag_2_SO_4_, of which 20 mL were distilled with 0.2 g of magnesium oxide. The distillate was collected in flasks containing boric acid and the indicators methyl red and bromocresol green, and then titrated with a standard 0.005 mol L^-1^ H_2_SO_4_ solution. The control followed the same procedures described above, but the urea was added after the addition of the KCl and Ag_2_SO_4_ solution.

### 2.8 N content in the beans, N content in the leaves, and N exportation by the beans

The N content in the beans and leaves were determined by the Kjeldhal method (Kjeldahl, 1883) after sulfuric digestion (Bremner, 1996). Samples of coffee beans were also collected from nearby commercial farms of the same age as the experimental area. These samples belonged to 8 different cultivars, commonly planted in the region. The beans were dried and processed to quantify the bean/husk ratio and the respective N content, according to the international NIST standard. This procedure allowed the comparison between the proportion of beans/husks and the exported amount of N of the main coffee cultivars, which is a piece of information currently unavailable in the literature.

### 2.9 Yield

The yield of the coffee plants was also evaluated in both crop seasons by manually harvesting the fruits when approximately 70% of them were mature (cherry stage). The harvested volume was measured, and a 5 L sample was sun-dried until reach 11.5% humidity. After drying, the sample was processed, weighed, and subsequently converted into 60 kg bags of processed coffee per hectare.

### 2.10 Calculations and statistical analyses

Data were subjected to a non-linear regression analysis using a logistic model (Equation 2) to evaluate the variable ammonia loss through volatilization. This model is already well-used for estimating plant growth, but it has been recently used to estimate the accumulated N-NH_3_ loss (Soares et al., 2012; Silva et al., 2017; Minato et al., 2020).


Equation 2 $$\text{Yi}=[\alpha /\{1+{\text{e}}^{\wedge }\text{ k}(\text{b}-\text{daai})\}]+\text{Ei}$$

Where Yi is the i-th observation of the cumulative loss of N-NH_3_ in %, being i = 1, 2… n; daai is the i-th day after fertilization; α is the asymptotic value that can be interpreted as the maximum cumulative amount of N-NH_3_ loss; b is the abscissa of the inflection point and indicates the day when the maximum loss due to volatilization occurs; k is the value that indicates the precocity index and the higher its value, the lower time needed to reach the maximum loss by volatilization (α); Ei is the random error associated with the i-th observation, which is assumed to be independent and identically distributed according to a normal distribution with mean zero and constant variance, E ~ N(0, I σ^2^).

To estimate the maximum daily loss (day of the maximum loss of N-NH_3_), that is, to determine the inflection point of the curve, the following equation was used:


Equation 3 MDL=k×(α/4)

Where k is a relative index used to obtain the maximum daily loss (MDL), and α would be the asymptotic value that can be interpreted as the maximum cumulative loss of N-NH_3_.

After checking for normality (Shapiro-Wilk’s test) and homogeneity of variances (Bartlett’s test), an analysis of variance was applied to test the influence of the N sources applied on the variables of N-NH_3_ loss by volatilization, urease activity, N concentration in the leaves, and yield. The significance of the differences was evaluated at *P* ≤ 0.05, and after validation of the statistical model, means were grouped by the Scott-Knott’s algorithm using R software 3.3.1 (R Development Core Team, 2018).

## 3 Results

### 3.1 Weather conditions

The accumulated precipitation on the 1^st^, 2^nd^, and 3^rd^ applications of the N fertilizer in the 2019/2020 crop season were 289, 457, and 336 mm, respectively, totalling 1082 mm. In the first seven days after each fertilization, the precipitation corresponded to 0 mm (0%), 63 mm (14%), and 159 mm (47%).

In the 2020/2021 crop season, the accumulated precipitation on the 1^st^, 2^nd^, and 3^rd^ fertilization were 291, 280, and 336 mm, respectively, totalling 907 mm. In the first seven days after each fertilization, the precipitation corresponded to 14 mm (15%), 28 mm (10%), and 0.2 mm (0.05%). The precipitation influenced the processes related to N-NH_3_ losses and we observed that lower rainfall in the first seven days after fertilization resulted in lower N-NH_3_ losses.

The area under the coffee canopy, where liming and fertilizers are applied, received 30 to 40% less precipitation compared to the inter-row area, depending on the rainfall volume and intensity, plant architecture, leaf area, and development stage.

The relative air humidity was above 70% for most of the period following the N fertilization in both crop seasons. The average temperature during the same period was 22 °C, with a minimum of 18.3 °C and a maximum of 36.6 °C.

### 3.2 N volatilization and urease activity

#### 3.2.1 Daily and cumulative losses of N-NH_3_

Daily and cumulative losses of N-NH_3_ from the three fertilizer applications in each crop year were influenced (*P* ≤ 0.05) by the N fertilizer technologies. The volatilization of N-NH_3_ was similar for the doses of 150 and 400 kg ha^-1^ of N. The application of U resulted in losses of 22.0% and 22.8% for these respective doses, considering the average of the two evaluated crop seasons. This means that 66 and 182 kg ha^-1^ of N were lost, respectively, at the end of six fertilizations with conventional urea (**Table 1**). 

**Table 1:** Mean Cumulative losses of ammonia (% of the applied N), for conventional and stabilized N fertilizers, in three fertilizations in the coffee plantation, during the 2019/2020 and 2020/2021 crop seasons.

Treatment	Dose (kg ha^-1^)	Ammonia Loss (%)								Mean*	LRCCU**	N-NH_3_ Loss	Avoided N-NH_3_ Loss
		2019/2020 Season				2020/2021 Season							
		1^a^	2^a^	3^a^	Mean	1^a^	2^a^	3^a^	Mean	(%)		kg ha^-1^	
Urea	150	19.8	12.6	14.6	15.7	20.2	29.4	35.4	28.3	22.0	–	66.1	–
Urea + NBPT		14.3	8.7	9.9	11.0	12.3	27.7	28.4	22.8	16.9	23.3	50.7	15.4
Ammonium nitrate		0.6	1.3	0.7	0.8	3.6	3.1	2.8	3.2	2.0	90.9	6.0	60.0
Urea	400	19.16	13.6	12.58	15.1	21.98	36.49	33.01	30.5	22.8	–	182.4	–
Urea + NBPT		17.17	10.17	7.49	11.6	18.08	31.63	34.08	27.9	19.8	13.3	158.2	24.3
Ammonium nitrate		0.74	0.28	0.25	0.4	1.19	1.51	1.73	1.5	1.0	95.8	7.6	174.8

NBPT, N-(n butyl) thiophosphoric triamide. In each crop season, 150 or 400 kg N ha^−1^ per year were split into three equal applications for conventional and stabilized N fertilizers, totaling 300 or 800 kg N ha^−1^ for both crop seasons, respectively. *Means followed by the same lowercase letter in the column do not differ by the Scott-Knott’s test (P ≤ 0.05). Mean of the six fertilization sources performed between November and February during both seasons. ** (LRCCU) Loss reduction compared to conventional urea.

U_NBPT_ reduced N-NH_3_ losses compared to U. Average losses of 16.9% and 19.8% were observed for doses of 150 and 400 kg ha^-1^ of N supplied by this stabilized source with a urease inhibitor additive. This reduction in volatilization prevented the loss of 15.9 and 24.3 kg ha^-1^ of N, respectively, compared to U (**Table 2**). As for AN, N-NH_3_ losses did not exceed 1% of the applied dose, regardless of the weather conditions at the time of the fertilization. This reduction in volatilization was 90.9 and 95.8% lower, respectively, preventing losses of 60 and 174.8 kg ha^-1^ of N compared to conventional urea (**Table 1**).

**Table 2:** Adjusted regression parameters for the accumulated and maximum daily losses of N-NH_3_ from conventional and stabilized N fertilizers in the 2020/2021 crop season.

Treatment	Split Fertilization	Crop season	Parameters				MDL
			α	b	k	R^2^	
			Maximum NH_3_ Loss	Day of the Maximum Loss			(kg)
–		2020/2021	**150 kg ha^-1^ of N**				
Urea	1		19.14	2.76	0.62	0.96	2.97
	2		28.79	1.91	1.23	0.99	8.85
	3		33.60	1.77	1.15	0.97	9.66
Ammonium Nitrate	1		3.52	9.49	0.45	0.99	0.40
	2		2.93	8.75	0.19	0.94	0.14
	3		2.64	8.24	0.21	0.97	0.14
Urea + NBPT	1		13.15	6.86	0.40	0.99	1.32
	2		25.62	3.48	0.71	0.97	4.55
	3		27.09	3.28	0.67	0.98	4.54
–			**400 kg ha^-1^ of N**				
Urea	1		21.48	4.07	0.58	0.99	3.11
	2		35.66	2.49	0.89	0.99	7.93
	3		32.26	3.08	0.73	0.99	5.89
Ammonium Nitrate	1		1.26	11.40	0.22	0.97	0.07
	2		1.29	4.30	0.23	0.89	0.07
	3		1.77	12.90	0.16	0.98	0.07
Urea + NBPT	1		17.83	5.08	0.57	0.99	2.54
	2		30.31	2.82	0.84	0.99	6.37
	3		33.09	4.14	0.50	0.98	4.14

α: Asymptotic value (percentage of the estimated maximum accumulated loss); b: Day when the maximum ammonia loss occurs; k: relative index; MDL (maximum daily loss of ammonia); and NBPT, N-(n butyl) thiophosphoric triamide.

In the 2019/2020 crop season, the maximum daily N-NH_3_ volatilization for U occurred 1.4 (7.36 kg ha^-1^ of N) and 1.88 (5.11 kg ha^-1^ of N) days after the application of 150 and 400 kg ha^-1^ of N. For AN, the maximum daily losses of 0.05 and 0.09 kg ha^-1^ of N occurred 7.31 and 5.76 days after the fertilization, respectively. For urea stabilized with NBPT, the maximum daily losses of 4.33 and 2.38 kg ha^-1^ of N occurred at 1.9 and 2.04 days after fertilization, respectively (**Table 3**).

**Table 3:** Adjusted regression parameters for the cumulative and maximum daily losses of N-NH_3_ from conventional and stabilized N fertilizers in the 2019/2020 crop season.

Treatment	Split Fertilization	Crop season	Parameters				MDL
			α	b	k	R^2^	
			Maximum NH_3_ Loss	Day of the Maximum Loss			(kg)
–		2019/2020	**150 kg ha^-1^ of N**				
Conventional Urea	1		19.66	3.87	0.57	0.99	2.80
	2		12.14	1.79	1.22	0.98	3.70
	3		14.29	1.40	2.06	0.99	7.36
Ammonium Nitrate	1		0.55	7.31	0.35	0.98	0.05
	2		1.28	4.59	0.15	0.97	0.05
	3		0.61	7.20	0.19	0.96	0.03
Urea + NBPT	1		14.15	5.64	0.62	0.99	2.19
	2		8.29	3.07	1.06	0.99	2.20
	3		9.57	1.90	1.81	0.99	4.33
–			**400 kg ha^-1^ of N**				
Conventional Urea	1		18.82	4.07	0.60	0.99	2.82
	2		13.16	2.55	1.00	0.98	3.29
	3		12.32	1.88	1.66	0.99	5.11
Ammonium Nitrate	1		0.73	5.76	0.51	0.98	0.09
	2		0.26	8.28	0.18	0.96	0.01
	3		0.23	6.90	0.21	0.96	0.01
Urea + NBPT	1		16.84	6.15	0.49	0.99	2.06
	2		9.81	3.46	0.78	0.98	1.91
	3		7.28	2.04	1.31	0.98	2.38

α: Asymptotic value (percentage of the estimated maximum accumulated loss); b: Day when the maximum ammonia loss occurs; k: relative index; MDL (maximum daily loss of ammonia); and NBPT, N-(n butyl) thiophosphoric triamide.

In the 2020/2021 crop season, the maximum daily loss for U occurred 1.77 and 3.11 days after the N fertilization, with 9.66 and 4.07 kg ha^-1^ of N, respectively. For AN, the maximum daily loss occurred 9.49 and 12.90 days after fertilization, with similar volatilization to the first season (less than 0.5 kg ha^-1^ of N). U_NBPT_ presented maximum losses at 3.48 and 2.82 days, with the volatilization of 4.55 and 6.37 kg ha^-1^ of N, respectively (**Table 2**).

The average cumulative loss of N-NH_3_ in the 2019/2020 and 2020/2021 crop seasons (considering the divided application of the N fertilizer) for the dose of 150 kg ha^-1^ of N decreased as follows: U (15.7%) > U_NBPT_ (11%) > AN (0.8%); and U (28.3%) > U_NBPT_ (22.8%) > AN (3.2%), respectively (**Table 1**).

Considering the dose of 400 kg ha^-1^ of N, the losses decreased as follows: U (15.1%) > U_NBPT_ (11.6%) > AN (0.4%); U (30.5%) > U_NBPT_ (27.9%) > AN (1.5%), respectively for the two crop seasons (**Table 1**).

During the first seven days, losses for the 150 kg ha^-1^ of N dose were: 14.4% > 9.08% > 0.48% (2019/2020) and 25.74% > 17.50% > 1.10% (2020/2021). For the 400 kg ha^-1^ N dose, these losses were 13.35% > 8.69% > 0.27% (2019/2020) and 26.96% > 22.42% > 0.61% (2020/2021). That is, for U, 88 to 92% of the cumulative N-NH_3_ losses occurred in the first week after fertilizer application in both crop seasons, while for U_NBPT_ accumulated losses ranged from 75 to 82%. For AN, on average, 50% of the cumulative volatilized N occurred in the first week, not exceeding 0.6% of the applied fertilizer dose (**Tables 3**, **2**).

#### 3.2.2 Urease activity

**Figure 4:**
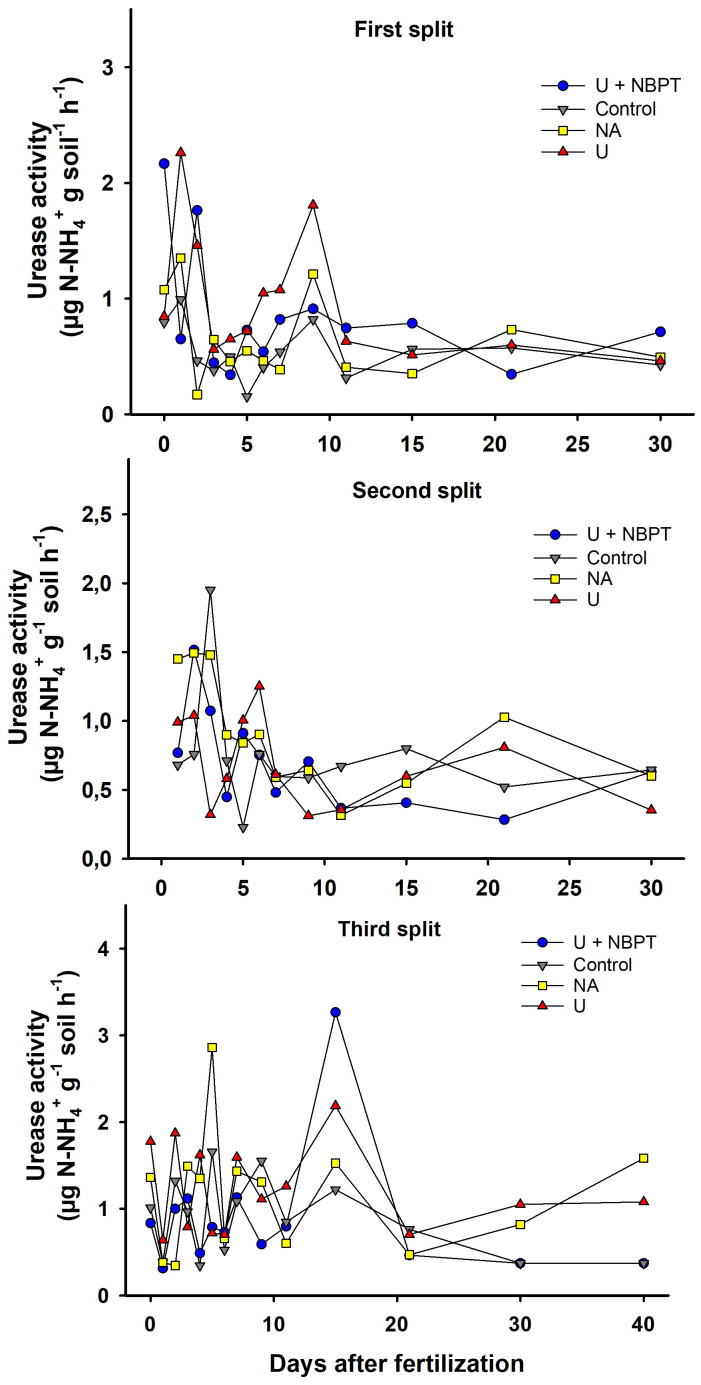
Soil urease activity after N fertilizer application with split fertilization (0 and 400 kg ha^-1^ of N) in coffee plantation.

Urease activity was higher on days of maximum NH_3_ volatilization. In general, the following sequence for the treatments was observed: U > U_NBPT_ > AN, despite some variations that are common for these factors (**Figure 4**). U_NBPT_ reduced the urease activity, proving that the additive associated with the fertilizer prolonged the urea hydrolysis due to the enzymatic inhibition that resulted in a decrease in N-NH_3_ losses presented in item 3.2.

### 3.3 Microbiological attributes

There was an effect of the N sources (NS), soil sampling time (ST), and their interaction (NS × ST) on the MBN and arylsulfatase activity. The N sources also influenced the MBC and the metabolic quotient (qCO_2_).

**Figure 5:**
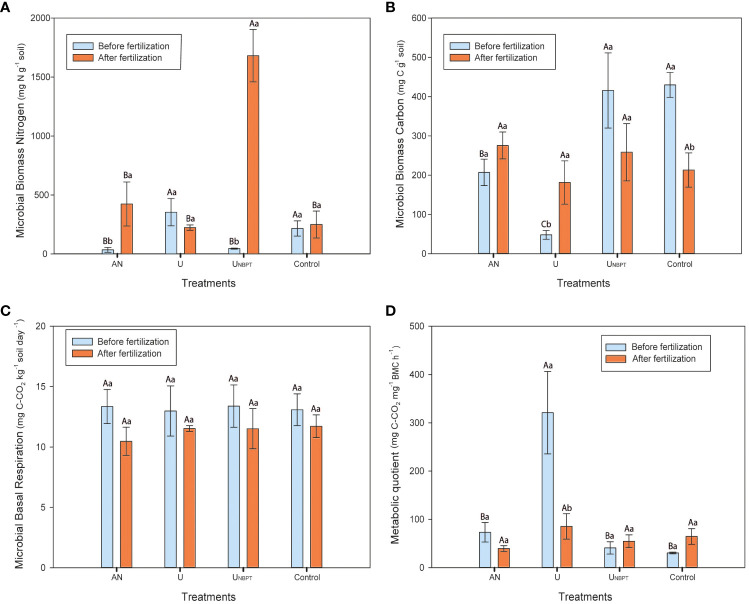
Microbial biomass nitrogen **(A)** and carbon **(B)**; soil basal respiration **(C)**; and metabolic quotient **(D)** as a function of the N sources (ammonium nitrate, conventional urea, urea + N-(n-butyl) thiophosphoric triamide (NBPT), and control without mineral N fertilization for six years), for the doses 0 and 400 kg ha^-1^ of N, and soil sampling time (samples 1 and 2). Means followed by the same uppercase letter between N sources and lowercase letter between soil sampling time do not differ statistically by Scott-Knott’s and F tests (P ≤ 0.05), respectively. Standard error bar; mean of four replicates.

In general, the soil showed high biological quality. The average content of N in the soil microbial biomass was higher after the N fertilization (average MBN = 644 μg N g^-1^ of dry soil), and the U_NBPT_ stood out (1681 μg N g^-1^ of dry soil) (**Figure 5A**). The MBN of this treatment before the fertilization (45 μg N g^-1^ of dry soil) was lower than the control without N fertilization (216 μg N g^-1^ of dry soil) and in the soil fertilized with conventional urea (354 μg N g^-1^ of dry soil). Soil microbial C content varied significantly with the N sources only before the fertilization (48 to 430 μg C g^-1^ of dry soil), where it decreased in the following order: U_NBPT_ = control > ammonium nitrate > conventional urea (**Figure 5B**). The sampling time did not interfere with the MBC in the control and urea + NBPT treatments.

The average SBR was 12 mg C-CO_2_ kg^-1^ dry soil h^-1^. The results of basal respiration of the N fertilizers remained equivalent to the control treatment in both sampling times (**Figure 5C**). Conversely, the soil fertilized with conventional urea had the highest qCO_2_ before the fertilization (**Figure 5D**). This result is directly related to the low microbial carbon identified in the initial sampling (48 μg C g^-1^ dry soil). The high qCO_2_ with conventional urea (321 μg h^-1^ C-CO_2_ μg^-1^ C-mic) represents about seven times the average of the other treatments for the same sampling time. However, this effect was not observed in the qCO_2_ after fertilization, where the result with conventional urea was statistically similar to the other treatments (**Figure 5D**), thus indicating the variability of these parameters.

**Figure 6:**
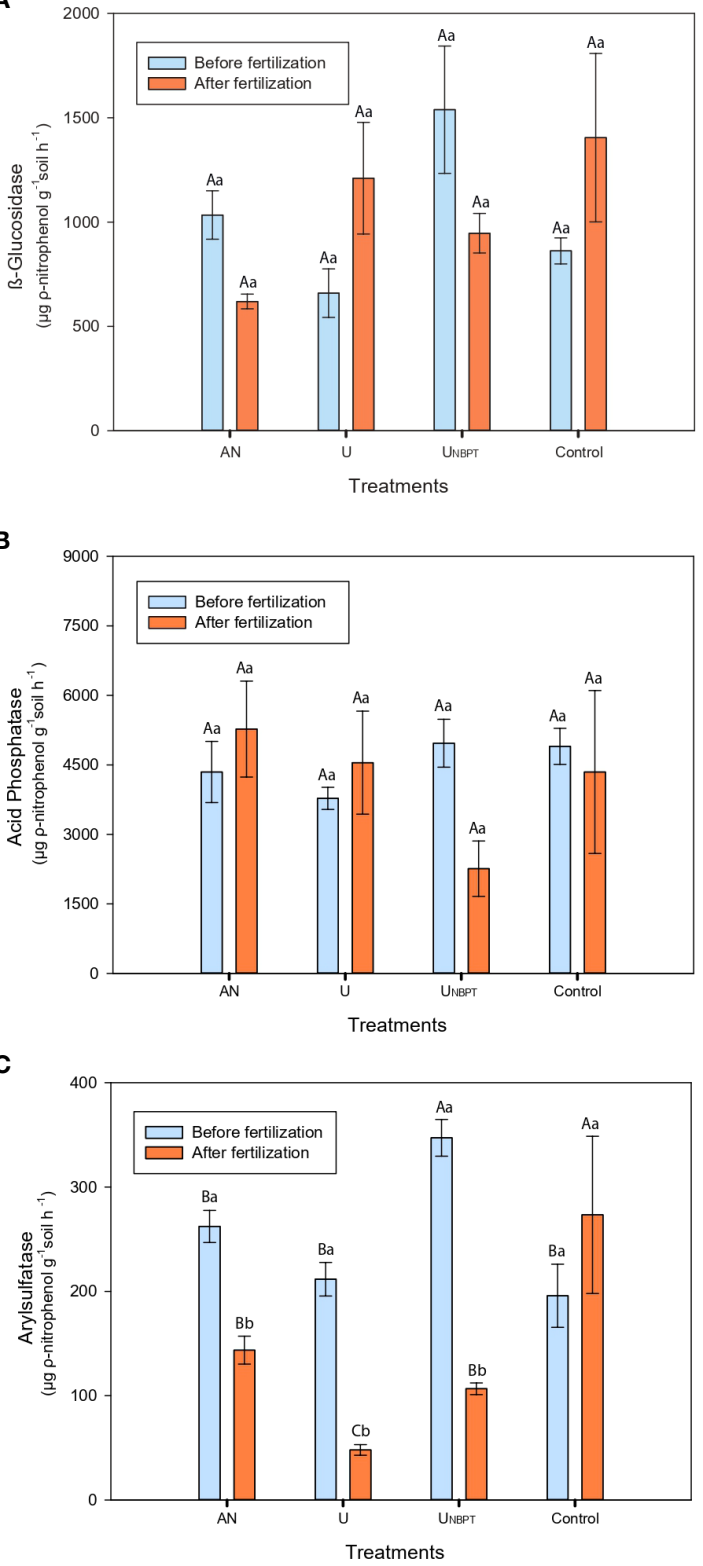
Enzymatic activity of β-glucosidase **(A)**, acid phosphatase **(B)**, and arylsulfatase **(C)** as a function of N sources (ammonium nitrate, conventional urea, urea + N-(n-butyl) thiophosphoric triamide (NBPT), and control with no mineral N-fertilization for six years), for the doses 0 and 400 kg ha^-1^ of N, and soil sampling time (sampling 1 and 2). Means followed by the same uppercase letter among nitrogen sources and lowercase letter among soil sampling time do not differ statistically by Scott-Knott’s and F tests (P ≤ 0.05), respectively. Standard error bars; mean of four replicates. PNP = ρ-nitrophenol.

The activities of β-glucosidase and acid phosphatase were not influenced by the addition of the N fertilizers in either of the soil sampling. High levels of p-nitrophenol (PNP), which reflect the enzymatic activity, were observed in both sampling times. For β-glucosidase (**Figure 6A**), PNP activity ranged from 3780 to 4970 µg PNP g^-1^ of dry soil before the fertilization and from 2260 to 4550 µg PNP g^-1^ of dry soil after the fertilization. For acid phosphatase (**Figure 6B**), PNP activity ranged from 660 to 1538 µg PNP g^-1^ of dry soil before fertilization and from 620 to 1405 µg PNP g^-1^ of dry soil after fertilization.

The arylsulfatase activity before fertilization was higher in the soil with NBPT, while the other N sources did not differ significantly in the first sampling time (**Figure 6C**). After fertilization, the control results were superior, especially compared to the conventional urea, which represented only 18% of the total activity of the treatment without N fertilization. There was a significant decrease in arylsulfatase activity between the samplings, with reductions of 45%, 69%, and 77% in the levels of p-nitrophenol (PNP) immediately after fertilization with ammonium nitrate, urea + NBPT, and conventional urea, respectively.

### 3.4 N content in the beans and leaves

The total N content in the beans of the 2019/2020 and 2020/2021 crop seasons were not influenced (*P* ≤ 0.05) by the isolated effect of the N doses and sources, and there was no significance (*P* ≤ 0.05) for the interaction between these factors (**Table 4**). The average N content was 2.6% in the beans and 1.6% in the husk, while the average bean/husks ratio is 1:1, despite the cultivar or productive load.

**Table 4:** N content in the beans (%) and N export per bag of processed coffee (kg N bag^-1^) for conventional and stabilized N fertilizers in a coffee plantation, during the 2019/2020 and 2020/2021 crop seasons.

Source	Dose	*N content in the bean (%)		Ept (kg N ha^-1^)		Ept (kg N bag^-1^)	
		2019/2020	2020/2021	2019/2020	2020/2021	2019/2020	2020/2021
**AN**	0	1.4	2.1	35.6	9.6	0.8	1.3
	150	2.3	2.6	87.6	21.8	1.4	1.6
	275	2.4	2.7	118.7	24.1	1.4	1.6
	400	2.6	2.5	148.2	20.5	1.6	1.5
	525	2.6	2.6	132.9	61.6	1.6	1.6
**U**	0	1.4	2.1	35.6	9.6	0.8	1.3
	150	2.4	2.4	78.5	26.1	1.4	1.4
	275	2.4	2.5	114.0	18.1	1.4	1.5
	400	2.6	2.4	135.6	22.7	1.6	1.4
	525	2.6	2.4	126.8	36.7	1.6	1.4
**U_NBPT_**	0	1.4	2.1	35.6	9.6	0.8	1.3
	150	2.3	2.5	108.2	19.0	1.4	1.5
	275	2.5	2.5	121.7	19.3	1.5	1.5
	400	2.5	2.6	118.1	25.3	1.5	1.6
	525	2.6	2.6	116.2	26.2	1.6	1.6

NBPT, N-(n butyl) thiophosphoric triamide; Ept: N export per bag of processed coffee. In each crop season, 150 or 400 kg N ha^−1^ per year were split into three equal applications for conventional and stabilized N fertilizers, totaling 300 or 800 kg N ha^-1^ for both crop seasons, respectively. *Means did not differ by the Scott-Knott’s test (P ≤ 0.05).

The average N content in the leaves (30 mg kg^-1^) of the samples collected during the green cherry stage was not influenced (*P* ≤ 0.05) by the isolated effect of the N doses and sources. There was also no significance (*P* ≤ 0.05) for the interaction between these factors (**Table 5**), which is explained by the high levels of N present in the soil.

**Table 5:** Percentage of beans and husks, and N content in the beans of different coffee cultivars at full productive potential, harvested during the 2020/2021 crop seasons.

	Coffee fruits		N content	
	Beans	Husks	Beans	Husks
**Cultivar**	**%**		**%**	
Bourbom Amarelo	53	47	2.6	1.6
Catiguá-MG2	44	56	2.5	1.4
Topázio Amarelo	50	50	2.4	1.4
Acaiá	43	57	2.7	1.9
Arara	47	53	2.6	1.8
Mundo Novo	49	51	2.8	1.6
Catuaí Amarelo	48	52	2.7	1.5
Catuaí 99	50	50	2.5	1.5
NIST standard	–	–	100	100

NIST, International standard for validation of foliar analysis.

The extraction and export of N by coffee plants depends on the crop’s productivity and vegetation intensity, which vary in accordance with the biennial nature of the crop and annual soil and climate conditions.

### 3.5 Yield

Treatments did not lead to different yields (*P* ≤ 0.05) in the 2019/2020 crop season. For the 2020/2021 crop season, there was a significant effect (*P* ≤ 0.05) of the N sources within the dose of 525 kg ha^-1^, with AN being higher than U and U_NBPT_ (**Table 6**).

**Table 6:** Coffee bean yield (bags ha^-1^) according to the interaction between the N sources and N doses during the 2019/2020 and 2020/2021 crop seasons.

Sources	Crop season/Doses (kg N ha^-1^)				
	0	150	275	400	525
	**2020**				
AN	42.4 A	63.5 A	82.4 A	95.0 A	85.2 A
U	42.4 A	54.5 A	79.2 A	86.9 A	81.3 A
U_NBPT_	42.4 A	78.4 A	81.1 A	78.7 A	74.5 A
	**2021**				
AN	7.65 A	14.00 A	14.87 A	13.64 A	39.47 A
U	7.65 A	18.10 A	12.08 A	15.75 A	25.46 B
U_NBPT_	7.65 A	12.64 A	12.88 A	16.19 A	16.82 B

AN, Ammonium nitrate; U_NBPT_, urea + N-(n-butyl) thiophosphoric triamide (NBPT). Uppercase letters cluster sources (with four replications each of each one of the doses) according to the Scott-Knott’s test.

In both seasons, no relationship was observed between the amount of N lost by volatilization and yield, meaning that yield was not affected even in the treatments that showed higher losses by volatilization (**Table 6**).

## 4 Discussion

### 4.1 Weather conditions, N-NH_3_ volatilization and urease activity

In this study, the climatic conditions, especially precipitation and temperature, directly influenced the losses of N-NH_3_ by volatilization. In both coffee seasons, most of the N-NH_3_ losses occurred during the first seven days after the split application of the N fertilizers. The precipitation during these first days was essential to incorporate the fertilizer into the soil and reduce the N-NH_3_.

Rainfall, depending on the volume and intensity, enables the incorporation of the N fertilizer into the soil (Dawar et al., 2011). Higher rainfall volume and intensity are usually associated with better fertilizer incorporation and, consequently, lower losses by volatilization. The lower precipitation volume immediately after the application of the fertilizers on the soil hindered the incorporation of the N and favored N-NH_3_ losses. Thus, our results indicated that relying solely on environmental conditions for fertilizer incorporation increases the risk of N losses and, consequently, lead to the low efficiency of the fertilization.

Moreover, the architecture of the coffee plant obstructs the direct incidence of rainfall that would incorporate the N fertilizer applied under the canopy. Additionally, the presence of plant residues on the soil also acts as a barrier to fertilizer incorporation and creates a favorable environment for volatilization due to the high concentration of urease. The low concentration of NBPT demonstrated by chromatography analysis, as mentioned, may often not be sufficient to inhibit urease activity, resulting in daily N-NH_3_ losses similar to those observed for conventional urea.

Ammonium nitrate is a widely used N fertilizer in Brazilian coffee cultivation. In this study, the use of this fertilizer reduced over 90% of the N-NH_3_ losses in both crop seasons. The insignificant loss of N-NH_3_ for this N source is related to its acid to neutral reaction in the soil, especially at pH< 7. Another positive aspect is that these fertilizers do not depend on weather conditions at the time of application (Freitas et al., 2022; de Souza et al., 2023). Therefore, ammonium nitrate can be a strategic option for N fertilization in coffee cultivation systems. Conversely, amidic N is reported as the most susceptible to N-NH_3_ losses and represents an average of 30% of the N applied in coffee cultivation areas in Brazil (Chagas et al., 2016; Dominghetti et al., 2016; Silva et al., 2017). This means that for every three fertilizer applications, approximately one is lost due to volatilization. For an average annual fertilization of 845 kg of urea per hectare (380 kg of N ha^-1^), 254 kg of urea per hectare (114 kg of N ha^-1^) are lost. This currently corresponds to a financial loss of US$ 228 per hectare (FL= average annual loss of N-NH_3_ x average value per kg N-fertilizer), considering US$897 as the recent average value per ton of fertilizer. For ammonium nitrate, these losses rarely exceed 1%, an amount that is probably emitted by the soil, which justifies the greater retention and storage of N in soil fertilized in the correct dose, time, and location.

### 4.2 Microbial and enzymatic activity

The soil microbiological attributes indicated high biological quality for this adequately fertilized scenario of productive coffee farming. The results demonstrate that soil sampling over time (before or after fertilization) can identify different levels of microbial activity, highlighting spatial and temporal variability for soil quality indicators. These indicators are not always related to the fertilization carried out in that crop season, but to the fertility management adopted over several years that favor microbial resilience and sustainability of the productive system.

These enzymes are related to the carbon (β-glucosidase) and phosphorus (phosphatases) biocycles and are an indicator of soil quality (Gil-Sotres et al., 2005; Moreira and Siqueira, 2006; Bastida et al., 2008).

The abundance of this enzyme in the soil is an indicator of a high rate of organic sulfur mineralization and high fungal biomass, which is the main producer of soil sulfate esters (Dick et al., 1996).

Some factors such as soil texture, organic matter, moisture, and oxygen affect the survival of microorganisms (Zantua and Bremner, 1977; Burns, 1982; Flores-Rentería et al., 2020) and, consequently, the level of microbial and enzymatic activity. Thus, soil biological quality indicators have little contribution to decision-making regarding short-term agronomic management of coffee crops, and the results reflect the management adopted for several years, necessary for significant changes to occur in C stocks (Sarkis et al., 2021), soil profile building for adequate plant development, organic residue input, and physical, chemical, and biological balance of the soil.

### 4.3 N content in the beans, N content in the leaves, and N exportation by the beans

The N content in coffee beans is a physiological characteristic of the plants themselves, independent of the cultivar (**Table 5**). The green cherry stage is the time of highest plant demand (Prieto Martinez et al., 2003) and this plants redistributes the stored N to the fruits, maintaining these levels almost constant, regardless of the productive load. In addition to the N exported by the processed coffee and the absorption necessary for plant growth (**Table 4**), a large amount of N is removed from the system by the husk of the harvested fruits, which represents approximately 50% of the harvested fruit weight (**Tables 4**, **5**). Is emphasized that the exceeding doses of N applied in coffee plantations results in a N_mineral_ movement from the surface to the subsurface (N loss by leaching) despite the source of the fertilizer (Sarkis et al., 2023). Cannavo et al. (2013) also observed that the addition of large amounts of N fertilizer every year in this system led to a soil N saturation and a high potential of N losses through NO_3_^-^.

### 4.4 Yield

In the experiment, the reduction in ammonia volatilization did not influence crop yield. Other authors reported that N volatilization, in the form of NH_3_, does not limit production (Chagas et al., 2016). This may be explained by the amount of N applied as fertilizer being more than sufficient for plant growth. The biennial cycle of coffee, with one year being more productive than the following, can make difficult the observation of the effects of treatments on yield. Furthermore, the N stock in the soil organic matter should be taken into account, as it can compensate for the N lost through volatilization in conventional fertilizers, providing a good short-term productivity for the crop. However, over time, there may be a reduction in the N stocks due to lower N retention in the soil. The lower N-NH_3_ losses from the use of AN can increase the average mineral N content by 50% when compared to urea and U_NBPT_ (Sarkis et al., 2021).

## 5 Conclusions

Our study showed that N fertilizer technologies are interesting options for reducing N-NH_3_ losses by volatilization, increasing N retention in the soil, and the sustainability of coffee production systems. U_NBPT_ inhibited urease activity in the soil and reduced N-NH_3_ losses compared to conventional urea, but it is not the most strategic option in the context of coffee farming because it depends on the volume and intensity of rain to incorporate the fertilizer after the fertilization. Regardless of the weather conditions, AN lost less than 1% of N by volatilization, being the safest option for N fertilization efficiency in coffee production. This characteristic is especially advantageous considering the specific microclimate in the projection of plant canopies that hinders the incorporation of the fertilizers by the direct incidence of rainfall. Thus, AN is the source that contributes the most to increasing soil N stocks and supplying the demands of the vegetative and reproductive stages of the coffee plants. Soil microbial and enzymatic activity indicated high biological quality for this appropriately fertilized productive coffee farming. Although indicators of soil biological quality have been widely discussed in recent scientific publications, our results indicated that these guidelines have done little to aid decision-making regarding agronomic management of coffee crop, reflecting the management practices adopted for several years that have resulted in the accumulation of organic carbon, development of soil profile for adequate plant growth, organic residue input and maintenance of physical, chemical, and biological balance of the soil.

## Data availability statement

The original contributions presented in the study are included in the article/**Supplementary Materials**. Further inquiries can be directed to the corresponding author.

## Author contributions

LS: Conceptualization, Data curation, Formal analysis, Investigation, Methodology, Writing – original draft, Writing – review & editing. MD: Conceptualization, Data curation, Formal analysis, Investigation, Methodology, Writing – original draft. DO: Conceptualization, Data curation, Formal analysis, Investigation, Methodology, Writing – original draft. TF: Conceptualization, Data curation, Formal analysis, Methodology, Writing – original draft. TS: Writing – review & editing, Conceptualization, Validation, Investigation, Resources. VRB: Writing – review & editing, Conceptualization, Validation, Investigation, Resources. DG: Conceptualization, Data curation, Funding acquisition, Investigation, Methodology, Project administration, Supervision, Visualization, Writing – original draft, Writing – review & editing.

## Supplementary material

Supplementary Figure 1 Photographs of the conventional fertilizers: urea **(A)**; ammonium nitrate **(B)**, and NBPT stabilized urea **(C)**.

## References

[B1] Adotey N. Kongchum M. Li J. Whitehurst G. B. Sucre E. Harrell D. L. (2017). Ammonia volatilization of zinc sulfate-coated and NBPT-treated urea fertilizers. Agron. J. 109, 2918–2926. doi: 10.2134/agronj2017.03.0153

[B2] Afshar R. K. Lin R. Assen Y. M. Chen C. (2018). Agronomic effects of urease and nitrification inhibitors on ammonia volatilization and nitrogen utilization in a dryland farming system: Field and laboratory investigation. J. Clean. Prod 172, 4130–4139. doi: 10.1016/j.jclepro.2017.01.105

[B3] Anderson T. H. Domsch K. H. (1993). The metabolic quotient for CO2 (qCO2) as a specific activity parameter to assess the effects of environmental conditions, such as pH, on the microbial biomass of forest soils. Soil Biol. Biochem. 25, 393–395. doi: 10.1016/0038-0717(93)90140-7

[B4] Aragão O. O. S. Oliveira-Longatti S. M. Caputo P. S. C. Rufini M. Carvalho G. R. Carvalho T. S.. (2020). Microbiological indicators of soil quality are related to greater coffee yield in the Brazilian Cerrado region Ecol. Indic 113, 106205. doi: 10.1016/j.ecolind.2020.106205

[B5] Azeem B. Kushaari K. Man Z. B. Basit A. Thanh T. H. (2014). Review on materials & methods to produce controlled release coated urea fertilizer. J. Control Release. 181, 11–21. doi: 10.1016/j.jconrel.2014.02.020 24593892

[B6] Bastida F. Zsolnay A. Hernandez T. Garcia C. (2008). Past, present and future of soil quality indices: a biological perspective. Geoderma 147, 159–171. doi: 10.1016/j.geoderma.2008.08.007

[B7] Bouyoucos G. J. (1951). A recalibration of the hydrometer method for making mechanical analysis of soils. Agron. J. 43, 434–438. doi: 10.2134/agronj1951.00021962004300090005x

[B8] Bremner J. M. (1996). “Nitrogen total,” in Methods of Soil Analysis. Part 3. Ed. Sparks D. L. (Madison: ASA), 1085e1121.

[B9] Burns R. (1982). Enzyme activity in soil: location and a possible role in microbial ecology. Soil Biol. Biochem. 14, 423–427. doi: 10.1016/0038-0717(82)90099-2

[B10] Byrne M. P. Tobin J. T. Forrestal P. J. Danaher M. Nkwonta C. G. Richards K.. (2020). Urease and nitrification inhibitors—As mitigation tools for greenhouse gas emissions in sustainable dairy systems: A review. Sustainability 12 (15), 6018. doi: 10.3390/su12156018

[B11] Cai H. Akiyama Y. (2017). Effects of inhibitors and biochar on nitrous oxide emissions, nitrate leaching, and plant nitrogen uptake from urine patches of grazing animals on grasslands: a meta-analysis. Soil Sci. Plant Nutr. 4, 405–414. doi: 10.1080/00380768.2017.1367627

[B12] Cannavo P. Harmand J.-M. Zeller B. Vaast P. Ramírez J. E. Dambrine E. (2013). Low nitrogen use efficiency and high nitrate leaching in a highly fertilized Coffea arabica–Inga densiflora agroforestry system: a 15N labeled fertilizer study. Nutrient Cycling Agroecosys. 95, 377–394. doi: 10.1007/s10705-013-9571-z

[B13] Cantarella H. Otto R. Soares J. Silva A. G. B. (2018). Agronomic efficiency of NBPT as a urease inhibitor: A review. J. Adv. Res. 13, 19–27. doi: 10.1016/j.jare.2018.05.008 30094079 PMC6077139

[B14] Chagas W. F. T. Guelfi D. R. Caputo A. L. C. Souza T. L. Andrade A. B. Faquin V. (2016). Ammonia volatilization from blends with stabilized and controlled-released urea in the coffee system. Ciênc. Agrotec. 40, 497–509. doi: 10.1590/1413-70542016405008916

[B15] Chien S. H. Prochnow L. I. Cantarella H. (2009). Recent developments of fertilizer production and use to improve nutrient efficiency and minimize environmental impacts. Adv. Agron. 102, 267–322. doi: 10.1016/S0065-2113(09)01008-6

[B16] Costa M. C. G. Vitti G. C. Cantarella H. (2003). N-NH_3_ losses from nitrogen sources applied over unburned sugarcane straw. Rev. Bras. Cienc. Solo 27, 631–637. doi: 10.1590/S0100-06832003000400007

[B17] Dawar K. Zaman M. Rowarth J. S. Blennerhassett J. Turnbull M. H. (2011). Urea hydrolysis and lateral and vertical movement in the soil: Effects of urease inhibitor and irrigation. Biol. Fertil. Soils 47, 139–146. doi: 10.1007/s00374-010-0515-3

[B18] Development Core Team R (2018). R: a language and environment for statistical computing (Vienna, Austria: R Found. Stat. Comput).

[B19] Dick R. P. Breakwell D. P. Turco R. F. (1996). “Soil enzyme activities and biodiversity measurements as integrative microbiological indicators,” in Methods for Assessing Soil Quality, Eds. Doran J. W. Jones A. J. (Madison: Soil Science Society of America), 247–272.

[B20] Dominghetti A. W. Silva D. R. G. Guimarães R. J. Caputo A. L. C. Spehar C. R. Faquin V. (2016). Nitrogen loss by volatilization of nitrogen fertilizers applied to coffee orchard. Cienc. e Agrotecnol. 40, 1–11. doi: 10.1590/1413-70542016402029615

[B21] Doran J. W. Parkin T. B. (1994). Defining and assessing soil quality,” In: Doran J. W. Coleman D. C. Bezdicek D. F. Stewart B. A. (eds). Defining soil quality for a sustainable environment, vol 35. (Madison, WI: Soil Science Society of America), pp 3–21. doi: 10.2136/sssaspecpub35.c1

[B22] European Committee for Standardization (2008) Fertilizers-Determination of urease inhibitor N-(n-butyl) thiophosphoric triamide (NBPT) using high performance liquid chromatography (HPLC). Available at: https://infostore.saiglobal.com/preview/is/en/2015/i.s.en1665.12015.pdf (Accessed August 2021).

[B23] FAOSTAT. (2021). Food and Agricultural Organization of the United Nations. Fertilizer by nutrients. Available at: http://www.fao.org/faostat/en/#data/RFN (Accessed 1 mar. 2022).

[B24] Flores-Rentería D. Sánchez-Gallén I. Morales-Rojas D. Larsen J. Álvarez-Sánchez J. (2020). Changes in the abundance and composition of a microbial community associated with land use change in a Mexican tropical rain forest. J. Soil Sci. Plant Nutr 20, 1144–1155. doi: 10.1007/s42729-020-00200-6

[B25] Freitas T. Bartelega L. Santos C. Dutra M. P. Sarkis L. F. Guimarães R. J.. (2022). Technologies for fertilizers and management strategies of N-fertilization in coffee cropping systems to reduce ammonia losses by volatilization. Plants 11, 3323. doi: 10.3390/plants11233323 36501362 PMC9741429

[B26] Gil-Sotres F. Trasar-Cepeda C. Leirós M. C. Seoane S. (2005). Different approaches to evaluating soil quality using biochemical properties. Soil Biol. Biochem. 37, 877–887. doi: 10.1016/j.soilbio.2004.10.003

[B27] Guelfi D. (2017). Fertilizantes nitrogenados estabilizados, de liberação lenta ou controlada. Informações Agronômicas IPNI 157, 1–14.

[B28] Heffer P. Prud’homme M. (2016). “Fertilizer outlook 2016–2020,” in International Fertilizer Association (ed.) 84th IFA Annual Conference, Fertilizer Outlook 2016-2020. (IFA: Moscow). 1–5.

[B29] Islam K. R. Weil R. R. (1998). Microwave irradiation of soil for routine measurement of microbial biomass carbon. Biol. Fert. Soils 27, 408–416. doi: 10.1007/s003740050

[B30] Kjeldahl J. (1883). Neue methode zur bestimmung des stickstoffs in organischen Körpern. Z. F. Anal. Chemie. 22, 366–382. doi: 10.1007/BF01338151

[B31] Lara-Cabezas A. R. Trivelin P. C. O. Bendassolli J. A. Santana D. G. Gascho G. J. (1999). Calibration of a semi-open static collector for determination of ammonia volatilization from nitrogen fertilizers. Commun. Soil Sci. Plant Analysis. 30 (3-4), 389–406. doi: 10.1080/00103629909370211

[B32] Martins M. R. Sarkis L. F. Sant’Anna S. A. C. Santos C. A. Araujo K. E. Santos R. C.. (2021). Optimizing the use of open chambers to measure ammonia volatilization in field plots amended with urea. Pedosphere 31, 243–254. doi: 10.1016/S1002-0160(20)60078-9

[B33] Minato E. A. Cassim B. M. A. R. Besen M. R. Mazzi F. L. Inoue T. T. Batista M. A. (2020). Controlled-release nitrogen fertilizers: characterization, ammonia volatilization, and effects on second-season corn. Rev. Bras. Ciênc. Solo. 44, 190108. doi: 10.36783/18069657rbcs20190108

[B34] Moreira F. M. S. Siqueira J. O. (2006). Microbiologia e Bioquimica do Solo. 2nd ed. (Lavras: Editora UFLA).

[B35] Prieto Martinez H. E. Scherrer Menezes J. F. Bartolomeu De Souza R. Alvarez Venegas V. H. Gontijo Guimaraes P. T. (2003). Critical nutrient ranges and evaluation of nutritional status in coffee-tree plantations of Minas Gerais. Pesqui. Agropecu. Bras. 38, 703–713. doi: 10.1590/s0100-204x2003000600006

[B36] Salamanca-Jimenez A. Doane T. A. Horwath W. R. (2017). Coffee response to nitrogen and soil water content during the early growth stage. J. Plant Nutr. Soil Sci. 180, 614–623. doi: 10.1002/jpln.201600601

[B37] Santos C. F. Aragão O. O. S. Silva D. R. G. Jesus E. C. Chagas W. F. T. Correia O. S.. (2020). Environmentally friendly urea produced from the association of N-(nbutyl) thiophosphoric triamide with biodegradable polymer coating obtained from a soybean processing byproduct. J. Clean. Prod. 276, 1–13. doi: 10.1016/j.jclepro.2020.123014

[B38] Santos C. F. Nunes A. P. P. Aragão O. O. S. Guelfi D. Souza A. A. Abreu L. B.. (2021). Dual functional coatings for urea to reduce ammonia volatilization and improve nutrients use efficiency in a Brazilian corn crop system. J. Soil Sci. Plant Nutr. 21, 1591–1609. doi: 10.1007/s42729-021-00464-6

[B39] Sanz-Cobena A. Misselbrook T. Camp V. Vallejo A. (2011). Effect of water addition and the urease inhibitor NBPT on the abatement of ammonia emission from surface applied urea. Atmos. Environ. 45, 1517–1524. doi: 10.1016/j.atmosenv.2010.12.051

[B40] Sarkis L. F. Andrade A. B. Guelfi D. Chagas W. F. T. Faquin V. Guareschi R. F. (2021). Carbon dioxide flux of conventional and slow or controlled release nitrogen fertilizers in coffee crop. Commun. Soil Sci. Plant Anal. 52, 2884–2897. doi: 10.1080/00103624.2021.1971690

[B41] Sarkis L. F. Dutra M. P. Santos C. A. Alves B. J. R. Urquiaga S. Guelfi D. (2023). Nitrogen fertilizers technologies as a smart strategy to mitigate nitrous oxide emissions and preserve carbon and nitrogen soil stocks in a coffee crop system. Atmosph. environ.: X Anal. 20, 100224. doi: 10.1016/j.aeaoa.2023.100224

[B42] Sha Z. Lv T. Staal M. Ma X. Wen Z. Li Q.. (2020). Effect of combining urea fertilizer with P and K fertilizers on the efficacy of urease inhibitors under different storage conditions. J. Soils Sediments. 20, 2130–2140. doi: 10.1007/s11368-019-02534-w

[B43] Silva A. G. B. Sequeira C. H. Sermarini R. A. Otto R. (2017). Urease inhibitor NBPT on ammonia volatilization and crop productivity: a meta-analysis. Agron. J. 109, 1–13. doi: 10.2134/agronj2016.04.0200

[B44] Snyder C. S. (2017). Enhanced nitrogen fertiliser technologies support the ‘4R’ concept to optimise crop production and minimise environmental losses. Soil Res. 55, 463–472. doi: 10.1071/SR16335

[B45] Soares J. R. Cantarella H. Menegale M. L. C. (2012). Ammonia volatilization losses from surface-applied urea with urease and nitrification inhibitors. Soil Biol. Biochem. 52, 82–89. doi: 10.1016/j.soilbio.2012.04.019

[B46] Soil Survey Staff (2018). Official Soil Series Descriptions (Washington, D.C: USDA-NRCS).

[B47] de Souza T. L. de Oliveira D. P. Santos C. F. Reis T. H. P. Cabral J. P. C. da Silva Resende É. R.. (2023). Nitrogen fertilizer technologies: Opportunities to improve nutrient use efficiency towards sustainable coffee production systems. Agric. Ecosyst. Environ. 345, 108317. doi: 10.1016/j.agee.2022.108317

[B48] Tabatabai M. A. Bremner J. M. (1972). Assay of urease activity in soils. Soil Biol. Biochem. 4, 479e487. doi: 10.1016/0038-0717(72)90064-8

[B49] Timilsena Y. P. Adhikari R. Casey P. Muster T. Gill H. Adhikari B. (2015). Enhanced efficiency fertilisers: a review of formulation and nutrient release patterns. J. Sci. Food Agric. 95, 1131–1142. doi: 10.1002/jsfa.6812 25043832

[B50] Vance E. D. Brooks P. C. Jenkinson D. S. (1987). An extraction method for measuring soil microbial biomass C. Soil Biol. Biochem. 19, 703–707. doi: 10.1016/0038-0717(87)90052-6

[B51] Zantua M. I. Bremner J. M. (1977). Stability of urease in soils. Soil Biol. Biochem. 9, 135–140. doi: 10.1016/0038-0717(77)90050-5

[B52] Zhang X. Zou T. Lassaletta L. Mueller N. D. Tubiello F. N. Lisk M. D.. (2021). Quantification of global and national nitrogen budgets for crop production. Nat. Food 2, 529–540. doi: 10.1038/s43016-021-00318-5 37117677

