# Supplementary figures and images for: Ammonia volatization from conventional and stabilized fertilizers, agronomic aspects and microbiological attributes in a Brazilian coffee crop system
Source: Front Plant Sci. 2023 Dec 8;14:1291662. doi: 10.3389/fpls.2023.1291662 (PMC10739428; doi:10.3389/fpls.2023.1291662)

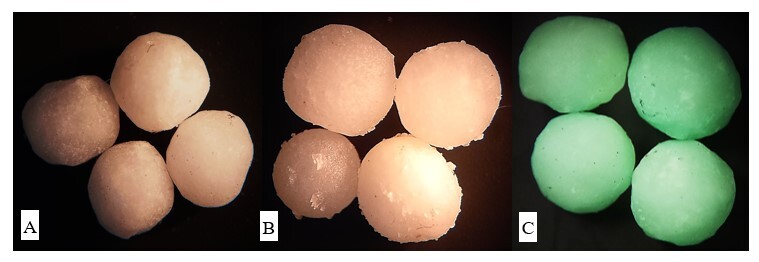

Supplement: Supplementary Figure 1 — Photographs of the conventional fertilizers: urea (A); ammonium nitrate (B), and NBPT stabilized urea (C). [file Image_1.tiff]
